# Evolutionary origins of non-adjacent sequence processing in primate brain potentials

**DOI:** 10.1038/srep36259

**Published:** 2016-11-09

**Authors:** Alice E. Milne, Jutta L. Mueller, Claudia Männel, Adam Attaheri, Angela D. Friederici, Christopher I. Petkov

**Affiliations:** 1 Institute of Neuroscience, Henry Wellcome Building, Newcastle University, Framlington Place, Newcastle upon Tyne, NE2 4HH, United Kingdom; 2 Centre for Behavior and Evolution, Henry Wellcome Building, Newcastle University, Framlington Place, Newcastle upon Tyne, NE2 4HH, United Kingdom; 3 Department of Neuropsychology, Max Planck Institute for Human Cognitive and Brain Sciences, 04103 Leipzig, Germany; 4 Institute of Cognitive Science, University of Osnabrück, Albrechtstr. 28, 49076 Osnabrück, Germany; 5 Centre for Neuroscience in Education, Department of Psychology, University of Cambridge, Downing Street, Cambridge, CB2 3EB, UK

## Abstract

There is considerable interest in understanding the ontogeny and phylogeny of the human language system, yet, neurobiological work at the interface of both fields is absent. Syntactic processes in language build on sensory processing and sequencing capabilities on the side of the receiver. While we better understand language-related ontogenetic changes in the human brain, it remains a mystery how neurobiological processes at specific human development stages compare with those in phylogenetically closely related species. To address this knowledge gap, we measured EEG event-related potentials (ERPs) in two macaque monkeys using a paradigm developed to evaluate human infant and adult brain potentials associated with the processing of non-adjacent ordering relationships in sequences of syllable triplets. Frequent standard triplet sequences were interspersed with infrequent voice pitch or non-adjacent rule deviants. Monkey ERPs show early pitch and rule deviant mismatch responses that are strikingly similar to those previously reported in human infants. This stands in contrast to adults’ later ERP responses for rule deviants. The results reveal how non-adjacent sequence ordering relationships are processed in the primate brain and provide evidence for evolutionarily conserved neurophysiological effects, some of which are remarkably like those seen at an early human developmental stage.

Understanding the developmental (ontogenetic) and evolutionary (phylogenetic) roots of the human language system is of substantial importance. The developmental perspective addresses how the neurobiological system supporting language learning develops towards a mature human adult form^1^,^2^,^3^,^4^,^5^,^6^,^7^,^8^. The evolutionary view clarifies which aspects of the neurobiology of human communication can be related to those present in our primate evolutionary relatives^9^,^10^,^11^, which also determines the aspects that can be realistically modelled at the neuronal level in primate neurobiological models. The interface of these research fields is equally important but is rarely empirically explored outside of the realm of behavioral study^12^,^13^. As such, certain differences between brain processes in adult humans and animal models might turn out to be remarkable correspondences if the neurobiological processes in nonhuman animals are compared with those in infants who are at early language learning stages. The present study aims to address this epistemic gap by testing whether and which brain potentials, previously associated with the processing of complex (non-adjacent) dependencies between spoken syllable sequences in human adults and infants, are evident in nonhuman primates.

In humans, certain language-related processes develop in infancy and early childhood^14^,^15^. An important pattern of many grammatical structures in human language are so-called non-adjacent dependencies. These dependencies in their simplest abstract form can be described by a *non-adjacent* relationship of the form *AXB.* For instance, in an ‘artificial grammar’, *A* and *B* elements in a three syllable long sequence are in a predictable relationship; if certain *A* syllables appear in the sequence, they must be associated with particular *B*s. The *adjacent* transitions from particular *A*s and a large number of intervening *X* syllables, as well as from the *X* syllables to certain *B*s, are unpredictable. At the surface, non-adjacent dependencies are also present in language (such as, *she often smiles*), yet, natural language also has hierarchical dependencies that require syntactic knowledge to be applied.

Human infants evidence incidental learning of non-adjacent dependencies from early on simply through habituation to *AXB* sequences^15^,^16^,^17^,^18^. Adults can also learn such non-adjacent dependencies^19^, although adult learning benefits from clear perceptual cues that *A* and *B* elements are associated^20^,^21^,^22^,^23^ and thus may be less incidental or automatic than it is in infants. This is also suggested by observations that adults fail to learn non-adjacent dependencies under passive conditions in which infants are successful^17^,^24^.

Structured sequence learning has been suggested as a language precursor capacity and recent behavioral work with nonhuman animals using artificial grammar learning paradigms has been clarifying the different types of sequencing dependencies to which various species of animals are sensitive^9^,^25^,^26^,^27^,^28^,^29^. The behavioral evidence suggests that various nonhuman animals are sensitive to certain types of *adjacent* dependencies^28^,^29^,^30^,^31^ and there is accumulating evidence of sensitivity to *AXB non-adjacent* dependencies in several species of nonhuman primates^32^,^33^,^34^. While, sequence learning may have been a prerequisite for syntax learning, the two are not synonymous. A syntactic sequence and the underlying structure is determined by the word category (or label), such as noun (for noun phrase) and preposition (for prepositional phrase). Since infants develop this word category knowledge in their first year, many human studies use sequence processing tasks to investigate the prerequisites of language/syntax learning. These paradigms also allow for a more direct comparisons across species^34^,^35^.

Despite the behavioral evidence in human and nonhuman animals, neurobiological data is missing on how non-adjacent sequencing operations are processed in the brains of nonhuman primates. Moreover, given that both sequencing and language-related processes differentiate during human development, it is unclear whether and which processes in nonhuman animals might relate to those in human infants or adults. For example, in a recent electroencephalography (EEG) study of event-related potentials (ERPs) in the infant brain, mismatch responses (MMRs) were observed that are remarkably similar in form in response to either pitch oddball syllables (pitch deviant) or sequence order violations of a non-adjacent rule dependency (rule deviant)^17^. In the same study, adult humans did not evidence learning of the non-adjacent relationship during passive listening, only showing an MMR in response to sound pitch deviants. When given an explicit task requiring them to respond to rule deviants, those who learned the non-adjacent rule showed a more delayed negativity, an N2 component^36^, which may reflect greater cognitive engagement or less automaticity for processing certain sequencing operations in adults than infants^17^. The marked differences between the neural signatures of non-adjacent dependency processing in infants and adults not only suggest different cognitive processes but also possibly the involvement of different neural processing pathways^37^.

This study aimed to identify the neural signatures sensitive to non-adjacent dependencies in the nonhuman primate brain using EEG, which also allows direct comparison with EEG potentials associated with non-adjacent sequencing dependencies in the human infant and adult brain. Here, we adapted the EEG paradigm previously used with human adults and infants to identify ERPs in response to the processing of pitch deviants and non-adjacent rule deviants^17^. Monkey ERPs show remarkably similar early MMRs in response to the pitch and (non-adjacent) rule deviants, as has previously been reported in infants^17^. We also identify and discuss late positivities seen in the monkey and adult ERPs. This study reveals the evolutionary roots of non-adjacent sequencing operations in primate brain potentials and shows evidence for conserved processes, some of which are evident at an early human developmental stage.

## Results

**Figure 1:**
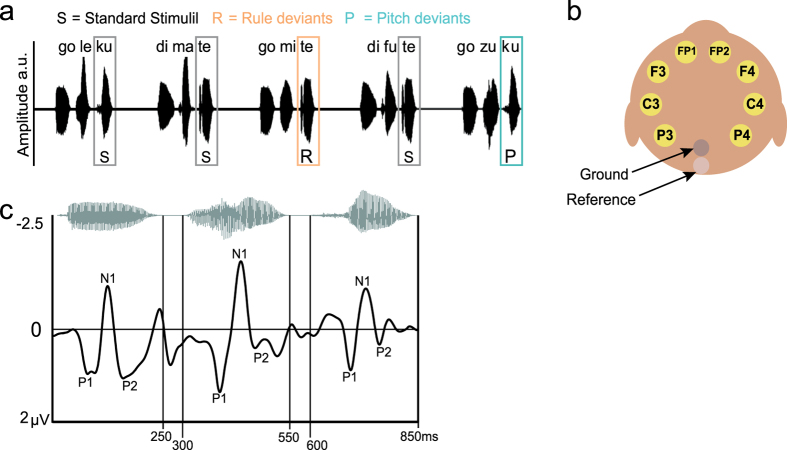
Exemplary triplet syllable sequences and monkey EEG electrode positions. (**a**) Audiograms of a series of standard (S) and pitch (P) or rule (R) deviant triplet syllable sequences. The first two sets of triplets illustrate standard sequences where the first and the third syllable are predictable (‘*go*’ is associated with ‘*ku*’; ‘*di*’ is associated with ‘*te*’), but the local transitions to and from the middle syllables do not predict the non-adjacent relationship. In the pitch deviant condition, (P) there was a ~11% increase in the pitch (F0) of the third syllable compared with the F0 averaged across all syllables. Pitch and rule deviants occurred independently. There were 20 different intervening elements: *ka, we, mi, no, gu, sa, me, ri, ro, ku, ma, ke, gi, ko, su, wa, xe, ki, so* and *mu* (sound amplitude in arbitrary units, a.u.), see: ref. 17. (**b**) Illustration of the approximate EEG electrode positions on the macaque head. (**c**) Grand average ERPs from all trials and all electrodes, stereotypical auditory ERP responses, P1, N1, P2 were observed for all three syllables (time after sounds onset: P1 = ~50 ms, N1 = ~100 ms, P2 = ~150 ms).

We recorded EEG signals as two Rhesus macaque monkeys listened to three syllable long sequences. Figure 1 shows the position of the EEG electrodes and illustrates the auditory sequences used for stimulation. Most of the sequences were *standard* sequences, where the voice pitch was consistent across all the syllables (i.e., the syllables were spoken by the same female speaker). Also all the standard sequences had *A* syllables in the 1^st^ position that were associated with specific *B* syllables at the 3^rd^ position in the sequences, including any combination of 20 possible intervening *X* elements in the 2^nd^ position (Fig. 1). Infrequent deviant sequences either had a voice-pitch oddball syllable (pitch deviant) in the 3^rd^ syllable position or a *B* syllable in the same position that violated the non-adjacent dependency between the *A* and *B* syllables (rule deviant, see Methods).

We evaluated the macaque EEG ERPs in response to each of the sounds in the sequence, observing stereotypical ERPs to each of the syllables in the sequence (see Fig. 1). To quantify the pitch- or rule-related effects, we analyzed the ERPs in response to the 3^rd^ syllable in the sequences, contrasting responses to the standard and deviant sequences. To determine which ERP components were statistically significant and consistent across the two animals, we used a multi-step procedure (Methods). Briefly, for each electrode and for each testing session, we subtracted the average waveform in response to the standard sequences from the deviant sequence response waveform to obtain a difference waveform. We then used the 500 ms silent baseline period preceding the sequences to calculate a non-parametric bootstrapped 95% confidence interval (CI) based on all 47 session (also see Attaheri *et al.*^38^, Methods). The CI was then projected onto the difference waveform for the third element. Where the difference waveform breached the CI for longer than 20 ms at time points that overlapped in at least four of the eight electrodes (50%), this was considered a component of interest. A session-by-session mean was calculated for each component based on the CI breaches across electrodes. An RM-ANOVA was used to assess the effects across electrode position (Laterality and Region) in relation to the effect of condition (Deviant vs Standard) with a between subjects factor of Macaque. Assumptions of normality for the use of the RM-ANOVA were met and all effects reported here were consistent for both animals. Further, we ensured that the mean values across electrodes submitted to a paired samples *t*-test for each animal were significant or were at least a statistical trend for each animal, adjusted for multiple comparisons (pitch and rule tests for each animal; corrected *p* value = 0.025).

### Pitch deviant ERP results

**Figure 2:**
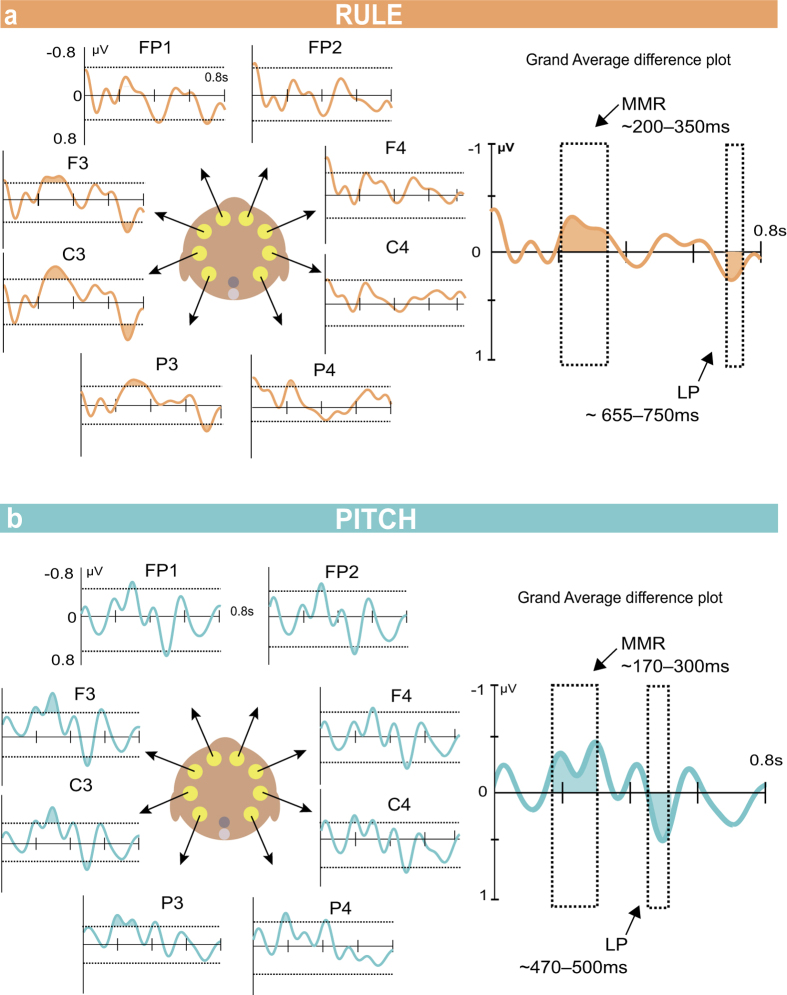
ERP components in response to pitch and rule deviants, by electrode. Conditions: (**a**) rule, (**b**) pitch. Shown are difference amplitude waveforms, Deviant minus Standard, for each of the electrodes. For each electrode a confidence interval (CI) was computed based on the 500 ms silent baseline period preceding sound presentation. Dashed horizontal lines represent the 2.5 and 97.5% CI boundaries. Where the waveform breached the CI, the timepoints during the breach were combined to create a single time window. The plots on the right show the grand average difference plot across all electrodes. The boxes indicated the significant components. In the rule condition there were two significant components, an MMR at 216–344 ms and a LP at 655–747 ms. For the pitch condtion there was an MMR at 159–316 ms and a LP at 469–515 ms.

Pitch deviant syllables elicited an early negative mismatch response (MMR) from 159–316 ms and a late positivity (LP) from 469–515 ms (Fig. 2). The RM-ANOVA confirmed the main effect of condition for both components (MMR: *F*_(1,45)_ = 64.35, *p* < 0.001; LP: *F*_(1,45)_ = 32.66, *p* < 0.001). In neither case did the effect of condition interact with animal, region (all *p*-values > 0.3), or laterality (*p* = 0.076). The main effect was also significant in each macaque separately (MMR Macaque 1 (M1): *t*_24_ = −5.37, *p* < 0.001; M2: *t*_21_ = −6.03, *p* < 0.001; LP M1: *t*_24_ = 3.934, *p* = 0.001; M2: *t*_21_ = 4.187, *p* < 0.001).

### Rule deviant ERP results

Two components of interest were present in the rule deviant waveform, an MMR at 216–344 ms and a LP at 655–747 ms (Fig. 2). The RM-ANOVA confirmed the effect of condition for both of these components (MMR: *F*_1,45_ = 7.990, *p* = 0.007; LP *F*_1,45_ = 8.23, *p* = 0.006). The MMR component showed no significant interactions with the other factors, including Macaque (*p*-values > 0.1). The main effect for the MMR was also significant in each animal separately (M1: *t*_24_ = −2.18, *p* = 0.039; M2: *t*_*21*_ = −2.54, *p* = 0.019).

For the LP there was a significant interaction between condition and laterality (*F*_1,45_ = 58.54, *p* < 0.001). Pairwise *t*-tests showed there was a significant positivity over left hemisphere electrodes (Right: *t*_46_ = −1.01, *p* = 0.317; Left: *t*_*46*_ = 5.34, *p* < 0.001). The main effect was confirmed in the left hemisphere for each animal (M1: *t*_24_ = 2.90, *p* = 0.008; M2: *t*_*21*_ = 5.08, *p* < 0.001).

### Comparison of early versus late testing sessions

**Figure 3:**
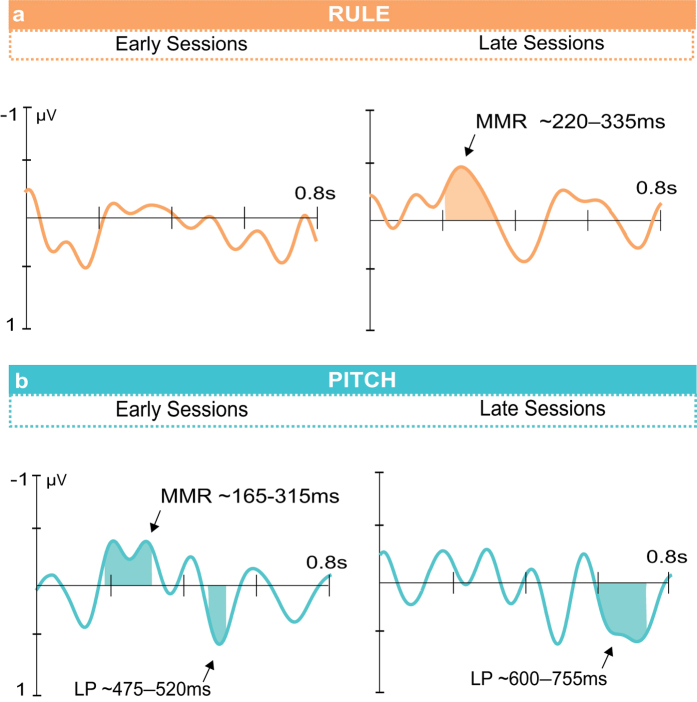
ERPs for early and late sessions. Data was collected over a number of testing sessions. Thus, it was possible to analyze effects over the early versus later testing sessions. In the rule condition (**a**), an MMR was evident in later sessions at 222–334 ms. A LP can be seen in the left hemisphere channels but did not reach the criteria for inclusion across all electrodes, see Fig. 2 and text. For the pitch condition (**b**) an MMR was present in the early sessions at 165–314 ms but no significant difference between early and later testing sessions was found. Two LP components were present for the pitch condition, one at 463–506 ms and another at 597–754 ms. Further statistical analysis of these showed no difference over time for the component at ~500 ms while the LP at ~700 ms was significantly stronger in the later sessions.

The macaques were tested over several testing sessions (>20 each). To determine whether the noted ERPs were evident in the first or second set of testing sessions, we divided the sessions into early and late testing sessions and evaluated the pitch- and rule-related ERP components within these split datasets (Fig. 3). Early sessions were based on the first 11 sessions for Macaque 1 (M1) and the first 12 sessions for Macaque 2 (M2), the last 11 and 12 sessions for each animal were combined to form the late testing sessions. The additional session 13 from M2 was excluded from this analysis to provide equal numbers of sessions in each group.

In the pitch condition the MMR was present in only the early sessions, occurring at 165–314 ms, but its amplitude did not show a significant difference between early and late sessions (see SI for details). For the rule condition, no ERPs were significant in the early testing sessions. In the later testing sessions for the rule, there was a significant rule MMR occurring at 222–334 ms, which was significant in both M1 and M2 (SI). For the rule, the RM-ANOVA showed a significant interaction of condition and region (Greenhouse-Geisser corrected: *F*_3,63_ = 3.14, *p* = 0.05). Further tests showed the MMR was stronger at frontal electrodes, particularly those over the left hemisphere in the later sessions (SI).

A late positivity (LP) also occurred in the pitch condition, in the early and later testing session (Early: 476–518 ms; Late: 463–506 ms; 597–754 ms) with no interactions between condition and electrode position (SI). Further analysis showed that the LP at 500 ms did not differ significantly between early and late sessions, whereas the LP at 700 ms was significantly more positive for later sessions. In the rule condition, the LP at 700 ms did not reached the inclusion criteria; using the time window from the overall main results showed no significant change between early and late sessions (see SI for details).

## Discussion

This study identifies macaque EEG brain potentials in response to pitch deviations in speech sounds and to non-adjacent dependencies between *A* and *B* syllables in *AXB* sequences. The results indicate that macaque brain potentials respond similarly to voice pitch deviants and non-adjacent dependency deviants, with both eliciting a mismatch response (MMR). We also found a set of macaque late positive (LP) ERP components in response to the two conditions. The results reveal first impressions on the neurobiological processes engaged by non-adjacent sequencing in the nonhuman primate brain. Moreover, the comparisons to human findings provide novel insights on how the human brain evolved for language. We separately discuss the ERP components observed in macaques in relation to those reported previously for the same sequencing relationships in human adults and infants^17^.

**Figure 4:**
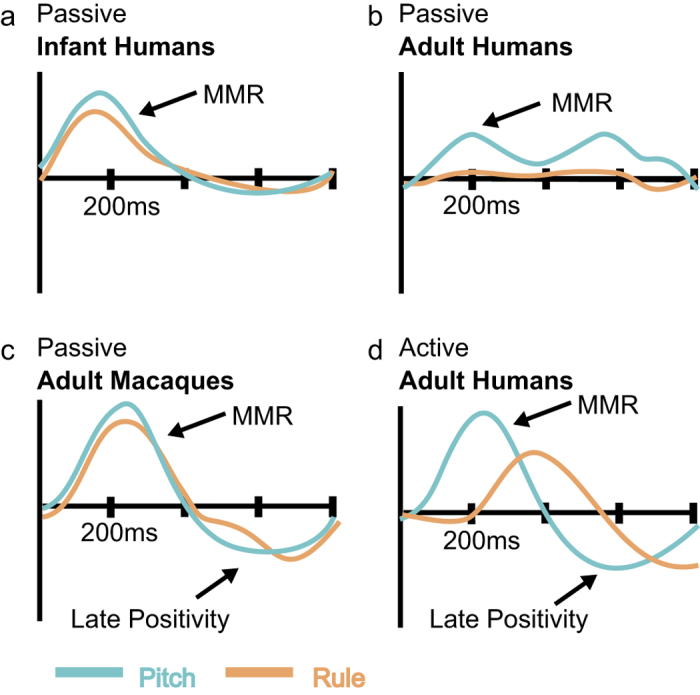
Schematized ERP components between species, human developmental stages and conditions. Human results schematized from Mueller, *et al.*^17^, depicting the mature ERP components from infants who acquired the non-adjacent rule with passive habituation (**a**), adult ERPs after passive exposure (**b**) and ERPs from adults that acquired the non-adjacent rule through an active task (**d**). The macaque ERP components illustrated are those found in this study in the late sessions (**c**). In macaques, an MMR was produced for the pitch deviant at ~200 ms. Only infants and macaques showed similar MMRs for the rule deviant as for the pitch deviant. In human adults no rule MMR is observed under passive conditions (**b**), and a negativity is observed in adults under the active rule conditions, evidenced as an N2 component that occurs later than the MMR (**d**). In human adult and macaque ERPs, later positivities are also observed for the rule and pitch conditions (**c**,**d**), which are not evident in the infant results (**a**). See text for discussion.

The macaque MMR elicited by non-adjacent dependency violations appears to share more similarities with the MMR for similar processes in infants than it does to the one in adults. The MMR occurs during a remarkably similar time window in both the macaque brain, as reported here, and in the infant brain as reported elsewhere^17^. Moreover, we found that the macaque MMR is elicited by the pitch deviant condition, consistent with other reports of the macaque mismatch negativity using oddball tasks^39^,^40^,^41^, and by violations of the rule sequencing relationship, as also seen with infants^17^. By comparison, human adults do not as readily implicitly glean non-adjacent relationships simply by habituation. Thus under passive stimulation conditions, such as those that the macaques and infants experienced, the adult EEG results tend to show an MMR for the pitch deviants but not for non-adjacent rule-deviants^17^. These effects are schematized in Fig. 4 for the three groups. Several behavioral studies have noted that many adults do not find non-adjacent dependencies obvious within ‘artificial grammar learning’ paradigms such as the one used here, unless the adults are provided with clear perceptual cues and/or an explicit task is used to direct them to the non-adjacent dependencies^19^,^21^,^22^,^23^,^28^. This suggests that in some cases, especially for more automatic rule extraction and speech discrimination, infants have an advantage over older individuals showing, for instance, better discrimination of foreign language phonemes^42^ and better learning of phonologically coded non-adjacent dependencies in a foreign language^24^. Adults that evidenced behavioral learning of the non-adjacent dependency in the EEG study (during an explicit task where they identify the sequences that follow or deviate from the non-adjacent rule dependency) show a different ERP component, evident as an N2 negativity that occurs later in time, see Fig. 4 and Mueller *et al.*^17^. Thus the macaque *AXB* rule-related effect particularly for the early MMR component is remarkably more like the one seen in human infants than adults. Moreover, we observed that the rule MMR, which was prominent in the later testing sessions, was stronger in left hemisphere central and frontal electrodes, a finding consistent with other mismatch studies (reviewed in Garrido *et al.*^43^), a previous macaque EEG study^38^, as well as during structured sequence learning in neonates^1^. By comparison, the pitch MMR was stable over the early and later sessions and did not show a strong topographical bias (Results).

In response to both the pitch and rule deviant conditions, we also observed a more positive later component for the deviant conditions. Late positivity (LP) ERP components were evident in the macaque results at ~500 ms post deviant onset for the pitch condition and at ~700 ms for the pitch and rule conditions (Figs 2 and 3). It is unclear whether the apparent differences in time of occurrence of the LP components reflect different processes, thus we refer to them simply as an LP component. In Mueller *et al.*^17^, a positive ERP component was observed in response to the pitch and rule conditions only in the adults (see schematic in Fig. 4). At least in human adults, this late positivity may involve or be related to the P3a, an ERP component seen with oddball paradigms^41^,^44^,^45^. As a point of reference, late ERP components for syntactic processes do not seem to appear in children until the age of 2.5 to 3 years^46^,^47^. In the nonhuman primates, the late positive ERP component that we label as a LP could be related to the reported P500 in macaques that responds to adjacent sequencing relationships^38^. As previously noted, the macaque P500 is unlikely to be a direct homolog of the P600 component in humans^38^, which by comparison in human adults is an EEG component associated with more complex forms of sequencing relationships^48^,^49^ or syntactic violations in natural language^50^. Since the LP is present in both the rule and the pitch conditions, its occurrence does not appear to be diagnostic for non-adjacent sequencing violations and may instead relate to other cognitive or mnemonic processes available to human adults and macaques but not infants. The lateralization pattern for the two potentials is however different. The LP at ~500 ms which is only present in the pitch condition shows no significant lateralization, while the LP at ~700 ms in the later testing sessions appears to be left lateralized in the rule condition.

Current theoretical ideas on the processes involved in structured sequence learning suggest that multiple distinct processes underlie sequence processing and learning. These depend also on the complexity of the sequencing operations. Namely, an implicit statistical learning process may support the sequence learning and rule extraction abilities of young children and infants. A more ‘top-down’ cognitive set of processes may develop and be in use at later states in humans^51^. The current results can inform these and other theoretical ideas, providing impressions on the neurobiological processes involved. A temporally early neurobiological set of processes appear to be engaged in both macaques and infants (Fig. 4). Macaques and adults show a later process that likely involves different sorts of operations, potentially for re-evaluating what was heard. Although there are a number of differences in the way that the monkeys on this study and the infants and adults in the reference study were tested (e.g., electrode coverage, task particulars), it is remarkable that amidst these differences there is nonetheless striking similarity in the monkey and infant MMRs and monkey and adult LPs. Yet a key difference in how the groups were tested relates to the use of an implicit task in the monkeys and infants and an explicit task with the adults. Thus, our impressions in macaques could benefit from being re-evaluated with an active task in the future to determine how an active task influences the observed ERP components during non-adjacent sequencing operations.

The observations of early MMR components in macaques and those previously reported in human infants support the notion that certain macaque early neurobiological processes may have been evolutionarily conserved and are present during early human developmental stages. It may be of interest to consider how the observed differences between macaques, human adults and human infants relate to key anatomical pathway variation between the groups. Alongside the functional developmental insights, we consider information on the differentiation of pathways of connectivity, such as the human dorsal arcuate fasciculus interconnecting temporal to frontal language regions^6^,^7^,^8^. Like adults, infants have a ventral pathway interconnecting the temporal lobe with inferior frontal cortex. However, the infant dorsal pathway seems mainly to project to premotor cortex, whereas in adults it more clearly projects to the posterior portion of Broca’s territory (Brodmann Area, BA44)^7^,^8^,^52^. The dorsal pathway is taken to be important for processing complex syntactic structures in adults, since development of the left arcuate fasciculus is associated with the improvement in performance with syntactically complex sentences in development from children to adults^37^. Although, structural connectivity data from non-human primates suggests that chimpanzees have a precursor of the dorsal arcuate fasciculus^53^, the extent to which monkeys have such a precursor tract, interconnecting auditory temporal lobe areas with ventral frontal cortex, remains unclear^54^,^55^,^56^,^57^,^58^. It is more broadly accepted that monkey auditory cortex interconnects with ventral frontal cortex by way of a ventral pathway or pathways^59^,^60^,^61^,^62^. Also, frontal regions along the ventral pathway (such as the frontal operculum) appear to be similarly sensitive to adjacent sequencing operations in humans and monkeys^63^, with the dorsolateral prefrontal cortex and associated dorsal pathways likely involved in other cognitive or mnemonic operations^64^,^65^. It is unknown which brain regions or pathways in non-human primates would be processing non-adjacent relationships. Thereby, the prior behavioral evidence suggests that both infants and monkeys are sensitive to *AXB* non-adjacent relationships, despite infants having a less well developed dorsal pathway and monkeys possibly also relying primarily on ventral pathways for certain sequencing operations.

Given that infants may have a less well developed dorsal pathway^7^,^8^ and monkeys may also possibly rely primarily on ventral pathways, at least for certain sequencing operations^63^,^64^,^65^, we hypothesize that non-adjacent sequence processes, especially those that elicit an MMR, may more strongly engage the ventral, rather than dorsal pathways in both human infants and macaques. Given also that in human adults the *AXB* rule dependency, when learnt, produces a later N2 rather than an MMR, this ERP component may depend on other pathways such as the dorsal pathway. The late positivity in macaques and human adults may depend on a more extensive set of brain processes. Future work using the current paradigm with a neurobiological technique better suited for localizing neurobiological responses, such as functional MRI in combination with diffusion-weighted structural connectivity^4^,^5^,^6^,^7^,^8^ could either support or disprove these hypotheses.

The comparative approach and the EEG results from this study lay a foundation for future empirical pursuits to directly identify which regions and pathways support the learning of complex sequencing dependencies. Eventually this could improve our understanding of which evolutionarily conserved processes are likely to have set a foundation for humans to crack the grammar code, illuminating the path that may have led towards language evolution in humans.

## Methods

Two adult male Rhesus monkeys (*Macaca mulatta*) from a group-housed colony were studied (Macaque 1, M1 = 15 years, 9.8 kg; Macaque 2, M2 = 7 years, 16 kg). All procedures performed were approved by the UK Home Office and comply with the Animal Scientific Procedures Act (1986) on the care and use of animals in research and also with the European Directive on the protection of animals used in research (2010/63/EU). We support the Animal Research Reporting of *In Vivo* Experiments (ARRIVE) principles on reporting animal research. As such, we used the minimum number of animals, consistent with the 3Rs principles, for which robust and repeatable results (from many testing sessions, see below) were obtained, in line with previous behaving macaque studies using two animals^40^,^41^. All persons involved in this project were Home Office certified and the work was strictly regulated by the U.K. Home Office.

### Stimuli

Stimuli were three syllable sequences; each syllable was separately recorded while spoken by a trained female speaker. All syllables used were 250 ms in length and presented with a 50 ms inter-syllable interval. The syllables were organized using Matlab scripts as depicted in Fig. 1 into standard, rule deviant and pitch deviant sequences. The stimuli were presented at ~75 db SPL (calibrated with an XL2 sound level meter, NTI Audio) using Cortex software, Salk Institute^25^.

### Procedure

The experiment used an oddball paradigm whereby a continuous stream of sequences was presented (inter-sequence interval = 650 ms). Each sequence constituted a testing trial: for each session 80% of the trials contained standard sequences and 10% of trials contained pitch deviants or 10% rule deviants. Stimulus sequences were presented in a pseudo-randomized order so that each first/third syllable pairing occurred with equal frequency. The sequence presentation also ensured that after each deviant, two, four, six or eight standard sequences had to occur before the next deviant sequence. If there were only two intervening standard sequences, we ensured that different deviant types occurred before and after.

### Time course of experiment

A fixation spot appeared in the center of the computer screen before the start of the first sequence and remained there for the full duration of the experiment. Each macaque’s task was to fixate on the spot and after approximately every 19 trials the macaque received a juice reward to maintain their motivation. The amount of reward was proportional to the time the animal spent fixating on the spot. The reward was delivered randomly and trials that occurred during reward delivery were removed. The length of the session was dependent upon the macaque’s fixation behavior and not all sessions produced usable data. In total, 22 usable sessions were completed by M1 and 25 by M2, generating 7,680 (M1) and 10,208 (M2) available trials for further analysis.

### EEG recordings and pre-processing

The experiment took place in an acoustically insulated and foam lined room (IAC). The macaque was seated in a custom made chair with the head immobilized via a central head post for EEG recording stability. EEG recording sessions typically took place five days a week although it was not always possible to test on consecutive days during the working week. Altogether the initial training and acclimatization of the animals to the laboratory environment and training on the eye-fixation task using juice rewards during EEG recordings required at least one year of work with each of the macaques; for further details see: ref. 54. The macaque was positioned ~60 cm in front of a monitor (24′′ Samsung, LCD). Each sequence was presented free-field from two audio speakers (Creative Inspire T10) placed horizontally at ±30° on either side of the monitor. EEG signals were recorded using eight Ag/AgCl (Silver/Silver-Chloride) electrodes held in place by a custom made cap (Fig. 1B). Additional details on electrode placement can be found in the SI. Signals were sampled at a rate of 1000 Hz through an EEG head stage and amplifier (Neuroscan). The reference was located at the back of the head (Fig. 1B). We used a low pass online filter set at 5000 Hz. Offline, the data were bandpass filtered between 0.3 Hz and 15 Hz. Noisy periods of data collection were manually identified and removed, blind to the experimental conditions. The remaining data were subjected to an independent-components analysis (ICA; ‘runica’ in EEGLAB) to further identify and remove artefacts. The data were then epoched from −500 ms to 1400 ms relative to the onset of the first element in the sequence and the waveforms were baseline corrected during the silent period between the sequences (from −500 to 0 ms, relative to sequence onset). For each session an average waveform was created per channel for each condition: pitch deviant, rule deviant, standards. This procedure was repeated for each macaque. The data were smoothed for graphical representation but not for analysis.

### Data Analysis

ERPs to pitch and rule deviants were analyzed separately. To assess the impact of a pitch deviant, we computed the average waveform across sessions for the standard condition and subtracted this from the average waveform across sessions for the pitch deviant condition. This resulted in a difference waveform. To identify where the difference waveform significantly differed from the standard waveform, we used the following multi-step procedure. Step 1: First, a non-parametric bootstrapped confidence interval (CI) was calculated for the difference waveforms during the 500 ms silent baseline. The 500 time points reflecting the silent baseline period preceding the sequence onset were sampled from each of the 47 sessions. We then computed a 95% bootstrapped confidence interval (1000 samples, using the bias corrected and accelerated method, BCA) for the mean of each of the 500 time points in the baseline period. The median values were calculated for each of the baseline confidence boundaries (97.5% and 2.5%), creating a CI that could be projected over later time points in the sequence (see SI). This was done separately for each electrode. Step 2: For each electrode, the time points where the difference amplitude exceeded the CI which were longer than 20 ms were identified to avoid overly brief breaches that can occur by chance. Step 3: Where the breaches overlapped in at least four of the electrodes (50%), this time period was treated as an ERP component of interest. The time windows used for later analysis were determined based on the first and last CI breach in the electrodes showing an effect of interest. Nearby breaches were combined into a single analysis TW if they remained above zero in the intervening period. Step 4: For each component, the average amplitude of each difference waveform during the TW was calculated on a session-by-session basis for each condition and for each electrode. These values were then subjected to a repeated measures analysis of variance (RM-ANOVA) to ensure statistical consistency in both animals. The RM-ANOVA had a between subject factor of Macaque (M1 vs. M2), a within-subjects factor of condition (Deviant vs. Standard) and factors defining the electrode position (region: frontal, fronto-central, central, parietal; hemisphere: left vs. right). A significant effect for macaque alone might be expected as a trivial difference in waveform amplitudes, which can occur for many reasons. Thus, any effect was only noted as being significant if it also showed consistency across the two animals, seen as a lack of a significant condition by macaque interaction. Furthermore for all components we conducted either a paired samples *t*-test or Wilcoxen signed rank test (when the data did not meet the normality assumption) for each macaque, to determine whether the effects were significant in both animals individually. To assess if the ERP components had changed over the first versus second half of the multiple testing sessions, we split the early and late testing sessions into two datasets. Since there was an odd number of sessions for M2 (25), the middle session was discarded; the first 11 (M1) and 12 (M2) sessions in each animal were assigned to the early condition and the final 11 (M1) and 12 (M2) to the late condition. We conducted the same previously noted statistical analyses separately for the early and late sessions to determine which effects were significant in the split datasets. For components of interest, we conducted an RM-ANOVA, taking the same time window from early and late sessions with an additional between-subjects factor of testing time (early vs late) and considered any interactions between condition and time. Where interactions occurred, these were investigated using paired-samples *t*-tests, adjusting for multiple comparisons.

## Additional Information

**How to cite this article**: Milne, A. E. *et al.* Evolutionary origins of non-adjacent sequence processing in primate brain potentials. *Sci. Rep.* **6**, 36259; doi: 10.1038/srep36259 (2016).

**Publisher’s note:** Springer Nature remains neutral with regard to jurisdictional claims in published maps and institutional affiliations.

## Supplementary Material

Supplementary Information
