# Supplementary material for: Evolutionary origins of non-adjacent sequence processing in primate brain potentials
Source: Sci Rep. 2016 Nov 9;6:36259. doi: 10.1038/srep36259 (PMC5101811; doi:10.1038/srep36259)
Supplement: Supplementary Information [file srep36259-s1.pdf]

SUPPLEMENTARY INFORMATION

To accompany: Alice E. Milne, Jutta L. Mueller, Claudia Männel, Adam Attaheri, Angela D. Friederici & Chris I. Petkov, *Evolutionary origins of non-adjacent sequence processing in primate brain potentials*

Electrode placement. The macaques studied had a central head post implant, which was used to immobilise the head for stability in EEG signal recording and eye-tracking measurements. These animals were also studied with functional MRI (Wilson et al., 2015, see manuscript references list). The EEG electrodes were placed around the implant on the top of the head over the skin (see manuscript Fig. 1). Unlike human EEG work, there is no universally accepted placement of electrodes on the macaque head. The human standard 10-20 system is used to guide the positioning of electrodes on the human head using physical features. The caps used in Mueller et al., 2012 followed the 10-20 system. In humans, the nasion (depression between the eyes) and the inion (a prominent bump at the base of the skull) act as two landmarks. Macaques possess both of these features and were thus used to establish landmarks for consistency in EEG electrode placement. The eight electrodes were then placed on FP1, FP2, F3, C3, P3, F4, C4, P4 locations (manuscript Fig. 1).

Bootstrapped confidence intervals: additional information. The experimental design is such that the three syllable sequences follow a rule structure of *AXB* where element *A*, is associated with element *B*. There are two *A* and *B* syllable pairs ("go" and "ku" as well as "di" and "te", see Fig 1). A deviant sequence occurs when element *B* either increases in pitch, or, if an incorrect *B* element is presented that would not in the standard sequences be associated with the A at the start of the sequence. When element *A* is heard, the animal in unaware of whether or not the sequence will contain a deviant *B* element. Additional analyses of the difference waveform over the *A* syllable provide a point of reference for the effects presented in the manuscript for the *B* item in the 3rd syllable position, as follows.

The manuscript Methods describe the statistical testing procedure. Briefly, event-related potential (ERP) components were only considered if they breached the bootstrapped Confidence Interval (CI) for more than 20ms, and the breaches occurred and overlapped in at least four (half) of the electrodes. Furthermore the component had to be consistent across both animals and significant or at least a trend in both of them.

In addition to this, we confirmed that the mismatch response (MMR) in the difference waveform was only exhibited in the presence of a deviant stimulus, as follows. Supplementary Fig. 1 shows the average baseline period for the difference waveform across each electrode and the testing sessions for the two conditions. The first 500ms is the baseline period before the start of the first *A* element in the sequence. The baseline period was extracted from each of the 47 sessions and used to generate the confidence intervals, as described in the manuscript Methods. Element *A* immediately follows the baseline period, but is not expected to elicit an MMR given that there are no deviant sounds present in the first syllable position in the sequences. In line with this, there is no clear evidence for an MMR in this period over which the CI are projected (see Suppl. Fig. 1 around 200ms). However, there are clear MMR potentials in response to the third element in the sequences containing the deviant stimuli (see manuscript Fig. 2). These supplementary analyses show that the CI projected to the 1st syllable in the sequences detect few differences beyond those that might be expected by chance, providing support for the use of the CI to detect effects elicited by deviant stimuli in the 3rd syllable, which is further assessed as described in the Methods.

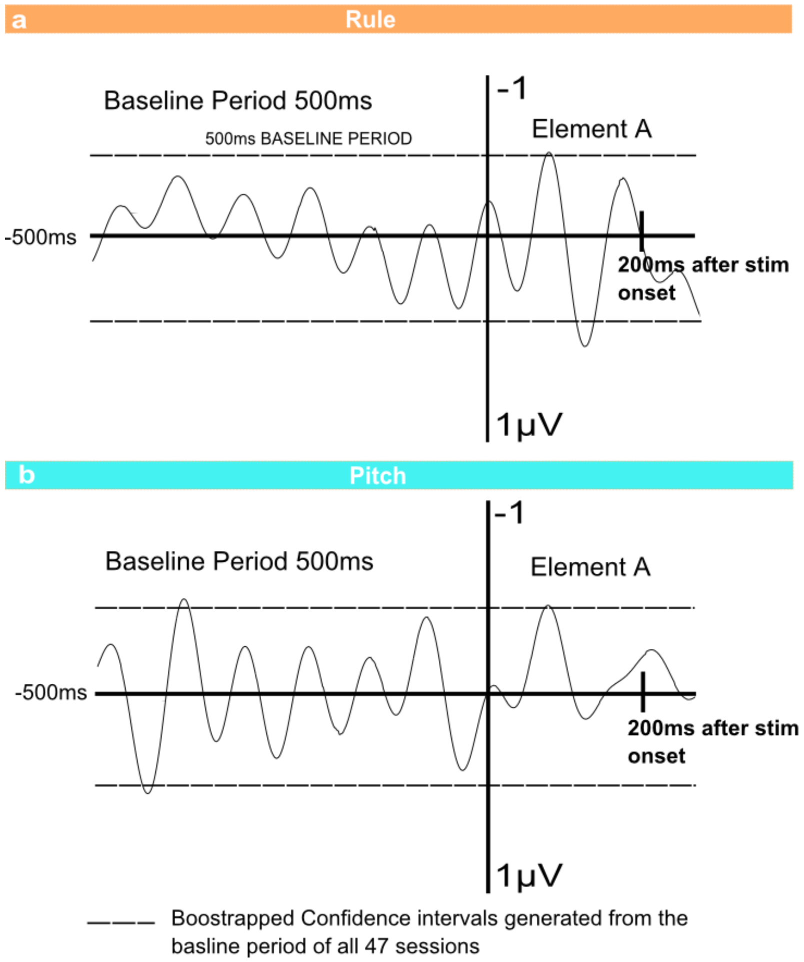

Supplementary Figure 1. Grand average difference waveform generated for the rule (a) and pitch (b) condition during the baseline and first *A* element periods. The baseline period shows the 500ms silent period before the start of the first sound. This time period was used to create a non-parametric confidence interval across sessions and the dashed lines represent the 2.5 and 97.5% confidence boundaries. Element *A* on the right of the figure shows the period during the first element. During this period an *A* syllable was presented but no deviant stimuli occur. For both conditions there is no breach of the confidence interval around 200ms, the time point at which a mismatch response is observed for the third element in the sequences, during which a deviant can occur (see manuscript, Fig. 2). Many of the breaches above or below the CI during the baseline and first item period are brief and could be expected to occur by chance.

Additional information on RM-ANOVA effects: Early versus Late effects

RM-ANOVA results are described here for the ERP components in the datasets from the early and later testing sessions.

Mismatch Response (MMR): In the early sessions there was an MMR component in the pitch condition between 165 to 314ms post stimulus onset. For this component, the RM-ANOVA showed a main effect of condition ($F_{1,176} = 21.43$, $p < 0.001$). There were no significant interactions between condition and the other factors. In the late sessions for the pitch condition the MMR component no longer reached the inclusion criteria, an independent samples *t*-test on the difference value for this time window across sessions demonstrated that the amplitude did not differ significantly between early and late sessions ($t_{44} = -1.21$, $p = 0.231$).

For the rule condition there were no time windows that reached the inclusion criteria for the early sessions, while for the late sessions there was an MMR at 222-334ms; the RM-ANOVA showed a main effect for the rule condition ($F_{1,21} = 20.69$, $p < 0.001$). There was also an interaction between condition and EEG electrode location (region), as reported in the main text, with no additional interactions. Paired-samples *t*-tests for each electrode showed that the two frontal electrodes and the left frontal-central electrode showed a significant effect of condition ($p < 0.005$), whilst posterior and right-side electrodes did not ($p > 0.05$). An independent samples *t*-test on the difference value for this time window across sessions demonstrated that the amplitude was significantly more negative in later sessions for the rule condition ($t_{44} = 2.39$, $p = 0.021$).

Late Positivity (LP): In the pitch condition there was a LP component in the early and later testing session at ~500ms (Early sessions: 476-518ms; Late sessions: 463-506ms) and at ~700ms in the late sessions (597-754ms). For each of these components, there was a significant effect of condition ($p < 0.002$), with no interactions. Independent samples *t*-tests showed no amplitude difference between the early and late sessions for the LP500 ($t_{44} = 0.518$, $p = 0.607$) while the LP at 700ms was significantly more positive in the later sessions for the pitch condition ($t_{44} = 3.88$, $p < 0.001$).

For the rule condition the split datasets did not show a significant LP across all electrodes, which might stem from the reduced power in these datasets and the rule LP being stronger in left hemisphere electrodes. Thus, to see if the rule LP was consistent between the early and later testing sessions, we checked this for the LP at 700ms in electrode C3. The results for this electrode, showing the strongest rule condition LP, showed no difference between early and late sessions ($t_{44} = 0.11$, $p = 9.14$).
